# Esophageal replacement in children - 27 years of experience in a University Hospital

**DOI:** 10.1590/0100-6991e-20243756-en

**Published:** 2024-07-05

**Authors:** FLAVIA GARCIA FROGERI, JOAQUIM BUSTORFF-SILVA, ANTONIO GONÇALVES DE OLIVEIRA, MARCIA ALESSANDRA CAVALARO PEREIRA-DA SILVA, THALITA MENDES MITSUNAGA, LUISA SARTI

**Affiliations:** 1 - Unicamp, Cirurgia Pediátrica - Campinas - SP - Brasil

**Keywords:** Esophagus, Esophageal Atresia, Esophageal Stenosis, Pediatrics, Esôfago, Atresia Esofágica, Estenose Esofágica, Pediatria

## Abstract

**Introduction:**

esophageal replacement in children is indicated when it is impossible to maintain the native esophagus, which in the pediatric population includes patients with esophageal atresia and esophageal caustic stenosis. The objective of this communication is to report the experience of a university service with two techniques of esophageal replacement.

**Methods:**

this is a retrospective study based on the revision of hospital files. The study population consisted of patients who underwent esophageal replacement from 1995 to 2022, at the Hospital de Clínicas of the State University of Campinas. The analyzed data were age, sex, underlying disease, technical aspects, complications, and long-term results.

**Results:**

during the study period, 30 patients underwent esophageal replacement. The most common underlying diseases were esophageal atresia (73.33%) and caustic stenosis (26.67%). Twenty-one patients underwent gastric transposition (70%), and nine underwent esophagocoloplasty (30%). The most frequent postoperative complication was fistula of the proximal anastomosis, which occurred in 14 patients. Most of the patients with fistulas had a spontaneous recovery. There were three deaths. Of the 27 survivors, 24 can feed exclusively by mouth.

**Conclusion:**

esophageal replacement in children is a procedure with high morbidity and mortality. Esophagocoloplasty and gastric transposition have similar results and complications, with the exception of proximal anastomotic fistulas, which are generally self-resolving and are more common in esophagocoloplasty. The choice of the best surgical technique must be individualized according to the patients characteristics and the surgeons experience, as both techniques offer the ability to feed orally in the short or medium term.

## RESUMO

**Introdução:**

a substituição esofágica em crianças está indicada quando não é possível manter o esôfago nativo, o que inclui principalmente pacientes com atresia esofágica e estenose cáustica esofágica. O objetivo deste trabalho é relatar a experiência de um serviço universitário com duas técnicas de substituição esofágica, a transposição gástrica e a esofagocoloplastia.

**Métodos:**

Estudo retrospectivo baseado na revisão de arquivos hospitalares. A população do estudo foi de 30 pacientes com idade entre 6 meses e quatorze anos, submetidos à substituição esofágica, no período de 1995 a 2022, no Hospital de Clínicas da Universidade Estadual de Campinas. Os dados analisados foram idade, sexo, doença de base, aspectos técnicos, complicações e resultados a longo prazo.

**Resultados:**

As doenças de base mais comuns foram atresia de esôfago (73,33%) e estenose cáustica (26,67%). Vinte e um pacientes foram submetidos à transposição gástrica (70%) e nove à esofagocoloplastia (30%). A complicação pós-operatória mais frequente foi fístula da anastomose proximal, que ocorreu em 14 pacientes. A maioria dos pacientes com fístula teve recuperação espontânea. Houve três mortes no total. Dos 27 sobreviventes, 24 conseguem se alimentar exclusivamente por via oral.

**Conclusão:**

A substituição esofágica em crianças é um procedimento com alta morbimortalidade. Esofagocoloplastia e transposição gástrica têm resultados e complicações semelhantes, com exceção de fístulas da anastomose proximal, que são em geral auto-resolutivas e mais comuns na esofagocoloplastia. A escolha da melhor técnica cirúrgica deve ser individualizada, sendo que ambas as técnicas oferecem a capacidade de alimentação via oral a curto ou médio prazo.

## INTRODUCTION

The first reports of esophageal replacement (ER) in children date back to the mid 50’s, and during the early years, the colon was the preferred intestinal segment to substitute the esophagus^1^ ^-^ ^3^. Despite the reports of other techniques for ER, like the gastric tube, gastric transposition, or jejunal interposition, to name a few, total gastric transposition gained popularity in the early 80s, following the publications of Professor Lewis Spitz, who reported good results with this technique^4^. 

The main indications for ER in the pediatric population are esophageal atresia, followed by caustic stenosis. Other conditions that may require ER are peptic strictures, esophageal achalasia, malignancies, congenital esophageal stricture, or other rare anomalies of the esophagus^5^ ^-^ ^12^. 

Until the late 1990s, the preferred operation for esophageal substitution in the Division of Pediatric Surgery of the Hospital de Clínicas of UNICAMP was esophagocoloplasty using the transverse-left colon as the conduit. In 1997, we started doing gastric pull-up due to the 

We reported here our experience with these two techniques of esophageal replacement in children, comparing them regarding technical aspects, functional results, and incidence of complications^13^ ^-^ ^14^. 

## MATERIALS AND METHODS

This is a retrospective study based on the revision of patient files from the hospital.

This study was submitted and approved by the Faculty of Medical Sciences research ethics committee at Unicamp (protocol number 68413723.0.0000.5404). All patients or caretakers were asked to sign an informed consent form.

### Patients

The study population consisted of 30 patients aged between 6 months and fourteen years who underwent esophageal replacement at the Hospital de Clínicas of the State University of Campinas (HC-Unicamp) between 1995 and 2022.

### Inclusion criteria

Patients who underwent esophageal replacement surgery by the Pediatric Surgery team at HC-Unicamp during that period.

### Exclusion criteria

Patients who underwent esophageal replacement surgery in another period or in another hospital or who refused to sign the informed consent form.

## METHOD

Information was collected from the medical records of the HC-Unicamp Medical Archives Service. The data collected consisted of age, sex, primary diagnosis, associated malformations, surgical technique, in addition to intra and postoperative complications, occurrence of cervical fistulas, time until complete oral feeding, duration of hospitalization, and medium and long-term outcome. We could find all these data in the patient’s medical records. 

### Gastric pull-up technique

The child is placed in the supine position under general anesthesia. A midline laparotomy plus closure of the previous gastrostomy (if present) is done. The greater curvature of the stomach is mobilized by dividing the short gastric vessels and the gastrocolic omentum, preserving the vascular arcades of the right gastroepiploic vessels. The lesser curvature of the stomach is freed by dividing the lesser omentum and ligating the left gastric vessels close to the stomach, taking care to preserve the right gastric artery. The lower esophagus is exposed by dividing the phrenoesophageal membrane, and, in the esophageal atresia cases, the distal esophagus stump is mobilized to the abdomen altogether with the stomach. In the case of ER due to caustic stenosis, esophagectomy is performed either by a digital trans mediastinal dissection or, when necessary, with the aid of a thoracoscopy or thoracotomy. A Heinecke-Mikulicz pyloroplasty is performed in all patients. The next step of the procedure is to prepare the stomach for its transposition by locating the highest part of the fundus and placing two stay sutures on its top. Attention is then shifted to the neck, and the existing cervical esophagostomy is mobilized approximately 4cm to allow an adequate anastomosis. A posterior mediastinal tunnel, the preferred pathway, is prepared by blunt dissection, both by the abdominal and cervical approach. The stomach is then transposed through the posterior mediastinum to the neck and is anastomosed to the proximal esophageal pouch in a single-layer interrupted suture after a 10-12 Fr trans anastomotic nasogastric tube is inserted. A jejunostomy is created to enable early enteral feeding, and a penrose drain is left close to the cervical anastomosis. 

### Colonic substitution technique

The child is placed in the supine position, under general anesthesia, and a midline abdominal incision is made. The colon’s vascular supply is carefully analyzed to determine which segment is best for transposition. The right colon is rarely used due to its inconstant blood supply. The transverse or left colon is preferred. Vascular clamps are placed in the middle or in the left colic artery to evaluate the graft perfusion. We prefer a combination of the middle and left colon by ligating the left colonic artery, preserving the middle colonic artery and the marginal artery system. The posterior mediastinal tunnel was prepared by blunt dissection, and the length of the colon needed for transposition was estimated. The colon is then divided, and anastomosis between the remaining colons is performed. In patients with caustic stricture, the esophagectomy must be performed, either by transhiatal approach or by thoracoscopy or thoracotomy. The cervical part of the operation is similar to the gastric pull-up technique described above. The distal esophagus is resected from the stomach by a linear stapling device, and the colon graft is transposed through the chest into the posterior mediastinum, taking care to pass the vascular supply behind the stomach so it is not compressed under the pylorus. The proximal anastomosis (between the esophagus and the colon) is created with a single layer of interrupted sutures, and the gastrocolic anastomosis is performed at the anterior gastric wall after trimming the distal part to prevent dilatation. A Heinecke-Mikulicz pyloroplasty is performed in all patients. A gastrostomy is left in place to enable early enteral feeding. A trans-anastomotic nasogastric tube is inserted, and a penrose drain is left close to the cervical anastomosis.

### Data analysis

Long-term survival and exclusive oral feeding were defined as primary outcomes. Intra- and postoperative complications were considered secondary outcomes. Results were tabulated and expressed as percentages for discrete variables and mean or median for numerical and continuous variables. Results were displayed in tables and graphs as needed. 

## RESULTS

### Demographic data and personal background

During the study period, 30 patients underwent esophageal replacement. Their ages ranged from 6 months to 14 years (the mean age was 53 months and the median age was 30 months). The majority of patients were male (56.67%).

 Thirteen patients (43.33%) had associated malformations, the most prevalent being heart disease (20%), followed by anorectal malformation (ARMF) and Down syndrome, both corresponding to 10%. It is important to emphasize that some patients had multiple malformations (Table 1). 

**Table 1:** General data of the 30 patients. (ARMF - anorectal malformation; UT - Urinary tract).

Data Background		Esophagocoloplasty	Gastric pull-up
Number of patients		9	21
Age		1-8 years	6 months-14 years
Gender	Male	05 (55.55%)	12 (57.14%)
	Female	04 (44.45%)	9 (42.86%)
Associated malformations	Heart disease	03 (33.33%)	03 (15%)
	ARMF	02 (22.22%)	01 (5%)
	Down syndrome	0	03 (15%)
	UT malformation	01 (11.11%)	01 (5%)
	Neuropathy	02 (22.22%)	0
	Duodenal atresia	0	01 (5%)
	Cryptorchidism	0	01 (5%)

### Type of operation and underlying diseases

Esophageal substitution was indicated in 22 patients (73.33%) due to long-gap esophageal atresia or failed attempts to primary anastomosis and in 8 patients (26.67%) due to caustic stenosis. 

**Figure 1:**
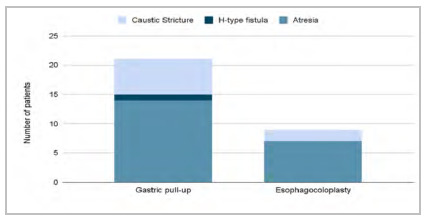
Types of operation and underlying diseases.

Gastric transposition was used in 21 patients (70%), and esophagocoloplasty in 9 patients (30%). The left colon was used in five patients and the right colon in four (Figure 1).

Seven patients had a history of previous esophageal operations, six of which were failed end-to-end esophageal anastomosis, and one was a failed esophagocoloplasty.

### Technical aspects

The most used route for graft transposition was the posterior mediastinum (63.33%), and the retrosternal route was used in cases with previous esophageal surgeries (36.67%). In the gastric pull-up group, the path used was the posterior mediastinum in 61.9%; in the colonic transposition group, this route was used in 66.7%.

The duration of the surgeries ranged from two to twelve hours, with an average of 362.85 + 141 minutes. The mean duration for both techniques was very similar, 369.9 + 96.2 minutes in the colonic transposition group and 372.8 + 158 minutes in the gastric transposition group.

Due to the need for esophagectomy, patients with caustic stenosis had a considerably longer surgical duration than patients who had an indication for surgery due to esophageal atresia (507.5+ 145.75 minutes vs. 308.42+ 99.1 minutes).

### Complications

The most common intraoperative complication was tension pneumothorax, which occurred in two patients submitted to gastric transposition. There was one case of mediastinal vascular injury during gastric transposition in a patient with caustic esophageal stenosis, which was promptly repaired with no adverse consequences. One child went into unexplained cardiorespiratory arrest while still in the operating room in the immediate postoperative period, and despite prompt assistance and cardiopulmonary resuscitation, the child developed permanent sequelae from hypoxic-ischemic encephalopathy.

Amongst the group that was subjected to gastric transposition, 13/21 (61,9%) children developed 20 complications. Amongst the patients subjected to esophagocoloplasty, 8/9 (88.8%) children developed 14 complications. 

Fistula of the proximal anastomosis was the most common postoperative complication, occurring in 14 patients (46.66%). Patients with esophageal atresia had a higher prevalence of cervical fistulas when compared to caustic stenosis patients (59.09% vs 12.5%; p<0.05) (Table 2). Also, a higher incidence of fistula and stenosis of the cervical anastomosis was observed in patients who had the colon as an esophageal substitute (66,7% vs 38,1%) (Table 3). The diagnosis of the cervical fistula was made 4 to 10 days after surgery, with an average of 6.58 days. There was spontaneous fistula closure in 13 patients (92.85%); in the remaining patients, the fistula was closed surgically. The mean time for fistula resolution was 19 days, except for one patient who persisted with a small amount of salivary discharge from the cervical surgical wound for 120 days. Six of these patients developed stenosis of the proximal anastomosis and needed variable periods of esophageal dilatation.

**Table 2:** Postoperative complications - comparison between underlying diseases.

Complications	Esophageal atresia	Caustic stricture
Number of patients	22	8
Fistula of the proximal anastomosis	13 (59,09%)	01 (12,5%)
Stenosis of the proximal anastomosis	06 (28,57%)	0
Evisceration	03 (14,28%)	0
Obstructive acute abdomen	0	01 (12,5%)
Chylothorax	02 (9,52%)	0
Splenic infarction	01 (4,76%)	0
Dehiscence of the gastric-colonic anastomosis	01 (4,76%)	0
Transposed colon ischemia	01 (4,76%)	0
Jejunostomy collapse	01 (4,76%)	0
Dumping	0	01 (12,5%)
Death	03 (13,63%)	0

**Table 3:** Postoperative complications - comparison between surgical techniques.

Complications	Esophagocoloplasty	Gastric transposition
Number of patients	9	21
Esophageal atresia/caustic stricture	7 / 2	15 / 6
Fistula of the proximal anastomosis	06 (66,66%)	08 (38,09%)
Stenosis	03 (33,33%)	03 (14,28%)
Evisceration	02 (22,22%)	01 (4,76%)
Obstructive acute abdomen	0	01 (4,76%)
Chylothorax	0	02 (9,52%)
Splenic infarction	0	01 (4,76%)
Dehiscence of the gastric-colonic anastomosis	01 (11,11%)	0
Transposed colon ischemia	01 (11,11%)	0
Jejunostomy collapse	0	01 (4,76%)
Dumping	0	01 (4,76%)
Death	01 (11,11%)	02 (9,52%)

### Postoperative follow-up

The duration of hospitalization ranged from 9 to 90 days, with a median of 20.5 days, and the time to start oral feeding ranged from 7 to 30 days, with a mean of 13.8 days. In the gastric transposition group, the length of hospital stay was 3 to 90 days, with a median of 19 days. This period was 12 to 60 days in the colonic transposition group, with a median of 15 days. Amongst the patients with esophageal atresia, the median length of hospital stay was 22,5 days, and amongst the patients with caustic stricture, it was 23,5 days.

Twenty-seven patients were available for late follow-up. The minimum follow-up time was two months, and the maximum was 190 months. The mean follow-up time was 67,7 months, and the median was 49 months. Twenty-four patients can feed exclusively orally, but four of them need periodic esophageal dilations. Two patients are fed both orally and by gastrostomy, and the patient with neurological sequelae, despite a patent gastric pull-up, receives diet only via gastrostomy.

There were two deaths in the gastric transposition group; one patient died in the immediate postoperative period due to refractory distributive shock after intense bleeding during the surgery, and another patient died of aspiration pneumonia nine months after the operation. In the colonic transposition group, there was one death 15 years after surgery due to bleeding from a gastrocolonic anastomosis ulcer. Therefore, there was only one post-operative death. 

 An additional child who suffered a cardiopulmonary arrest immediately after the operation, while still in the operating room, despite prompt resuscitation, progressed with neurological sequelae and remained bedridden since the operation. 

## DISCUSSION

The indications for esophageal replacement are esophageal conditions that prevent the native esophagus from being maintained. In the present series, this surgery was indicated mainly for patients with esophageal atresia without the possibility of primary anastomosis and for patients with caustic strictures refractory to esophageal dilations. Caustic lesions are caused by the ingestion of acidic substances, which cause coagulation necrosis, or alkalis, which cause liquefaction necrosis, which usually affects more profound layers of the esophagus. These types of injuries are still quite common in the pediatric population, most often being an accidental ingestion. Stenosis caused after caustic ingestion can be diagnosed after one month of the event, and strictures longer than 3 centimeters or multiple strictures usually do not respond well to esophageal dilation by endoscopy^7^ ^,^ ^9^ ^,^ ^12^. 

The ideal esophageal substitute for children should be a resistant conduit that will continue to function for many decades, that allows for adequate oral ingestion, without dysphagia, with minimal gastroesophageal reflux, and without causing compression of mediastinal structures. Based on these concepts, different surgical alternatives can be used depending on anatomical factors, previous surgeries, and the surgeon’s experience^7^ ^,^ ^9^ ^-^ ^12^ ^,^ ^15^. 

The esophagus can be replaced by a segment of the colon, stomach, jejunum, or gastric tube. The colon has good vascularization and an adequate diameter, and the most used segment is the transverse colon, based on the left colic artery. However, esophagocoloplasty includes the confection of three anastomoses: esophago-colon, colon-stomach, and colon-colon. Emptying of the transposed colon is exclusively by gravity, and the colon can become very dilated over time, causing dysphagia, slowed emptying, and stasis^7^ ^,^ ^9^ ^,^ ^12^ ^,^ ^16^ ^-^ ^18^. In our series, the right colon was used in approximately half of the patients who were submitted to esophagocoloplasty (4 out of 9 patients) due to vascularization of the colon and the possibility of mobilization into the neck. 

Transposition of a gastric tube, using a part of the greater curvature as a conduit, has been used with variable results because of its good vascular supply. However, the long suture line may predispose to fistulas and, in the long term, to gastroesophageal reflux and progressive dysfunction of propulsion^7^ ^,^ ^9^ ^,^ ^12^. 

Since the reports by Spitz and cols, gastric transposition, mobilizing the entire stomach through the mediastinum and constructing an anastomosis with the cervical esophagus, has been used more often in children. This operation is usually associated with a pyloroplasty due to the section of the vagus nerves during the procedure. To carry out the transposition, the left gastric artery, and the short vessels must be sectioned, preserving the remaining gastric irrigation. Anatomical studies have shown that gastric intraparietal vascularization is very rich and can maintain gastric viability. The presence of gastric mucosa adjacent to the cervical esophagus predisposes to a high incidence of gastroesophageal reflux, which must be monitored postoperatively. Although some authors claim that this would predispose to cervical esophageal cancer, this has not been clearly demonstrated in the literature^5^ ^,^ ^8^ ^,^ ^19^. The potential advantage of this technique over the gastric tube is the lower risk of fistula and stenosis^8^ ^-^ ^10^ ^,^ ^13^ ^-^ ^15^ ^,^ ^19^ ^-^ ^21^. 

The jejunum graft is the least used since an extended length of the intestine must be resected for its construction due to the layout of the vascular arcade of the jejunum, which has short vessels. Furthermore, the jejunum is less resistant to gastric acid secretion and may develop erosion of its wall and other complications. Therefore, it has yet to be considered as a first choice for esophageal replacement^7^ ^,^ ^9^ ^,^ ^12^. 

In recent years, we have been using the stomach as the primary esophageal substitute, leaving the colon as a second alternative depending on the patient’s history and the anatomical conditions during the intraoperative period. Even though this is not a randomized series, and these results have to be regarded in this context, our data could not show a clear superiority of one technique over the other. 

These data stress the importance of the surgeon involved with the treatment of these children being familiar with all the esophageal substitution techniques. In some situations, once the anatomy is evaluated, the decision of the best operation must be made after the beginning of the operation.

The esophageal substitute can be transposed to the cervical region for the anastomosis via the retrosternal route or via the posterior mediastinum (orthotopic position), the latter being preferred due to its shorter and more linear path. This path is particularly useful in cases of caustic stricture when the native esophagus should preferably be resected due to the high risk of malignancy (1.8 to 16%). The most demanding part of this operation is the blunt dissection of the posterior mediastinum, developed from below via the hiatus and from above through the cervical incision. Often, this dissection is made blindly and can be very hazardous, especially if the patient has had previous esophageal operations or, in the case of caustic strictures, when there may be firm adhesions between the esophagus and trachea. During this dissection phase, the surgeon’s fingers should remain always in contact with the spine to avoid trachea or aorta lesions. If firm adhesions are found due to previous surgery or esophageal perforation, early recourse to thoracotomy and dissection of the esophagus under direct view are recommended^15^. 

Regarding the ideal time to perform the surgery, in cases of indication due to esophageal atresia, it is recommended that the procedure be postponed until after the child has started to walk, as there is a higher mortality rate when the surgery is performed at an early age^22^. In our series, eight children were operated on before two years of age. Although a small number to allow for any statistical analysis, these children did not have more complications than the older ones.

Esophageal replacement is a complex surgical procedure and is not without risks. The most frequent complications are fistula and stenosis of the proximal anastomosis. Our series found a general incidence of fistula of the cervical anastomosis of 46.66%. Reports from other series indicate that the incidence of fistula varies from 17,6% to 36%. 

Overall, 70% of the operated children developed some kind of complication. The most common complication was fistula of proximal anastomosis.

Fistulas appears to be more common in patients submitted to esophagocoloplasty (66.66% vs 38,84%) and among patients operated for esophageal atresia (59.09% vs 12,5% p<0.05). Tannuri et al., also report a lower incidence of fistulas after gastric transposition than esophagocoloplasty. The lower incidence of fistulas in caustic stricture patients may be because the esophagectomy performed in children with caustic stricture leaves a larger tunnel in the posterior mediastinum than the one created in children with esophageal atresia. It is also possible that the higher incidence of fistulas in patients with esophageal atresia compared to those undergoing treatment for caustic stenosis is due to the fact that the former usually present with esophagostomy, while the latter are generally taken to surgery with the esophagus anatomically intact. In all but one child, the fistulas healed spontaneously without further interventions. It is of note, however, that almost half of the children who developed a fistula progressed with stenosis of the cervical anastomosis and needed variable periods of esophageal dilatation ^5^ ^-^ ^7^ ^,^ ^11^ ^,^ ^12^ ^,^ ^23^ ^-^ ^25^.

The most important limitation of this study is its descriptive nature, which makes inferences impossible. It is difficult to perform a case-control study on this subject, considering that children who need an esophageal replacement cannot be randomly placed in a group for a specific surgery since the choice of the technique depends on the background and anatomy of the patient. A prospective cohort study would allow us to compare both techniques.

## CONCLUSION

Esophageal replacement surgery is a complex and very demanding procedure, often followed by a turbulent postoperative period and associated with significant morbidity and mortality. Therefore, it must be performed in specialized centers where you can rely on a multidisciplinary team, including pediatric anesthesiologists, intensivists, radiologists, and also endoscopists, to promptly diagnose eventual complications and treat them as soon as possible. The choice of the best surgical technique should be individualized to the patients’ needs and according to the surgeon’s experience. Despite being a complex and demanding procedure, when performed by experienced hands and adequate facilities, it is associated with a high success rate (>80% in most series), measured by the ability of these children to feed normally in the long term.

## References

[1] Sherman CD, Waterson DJ (1957). Esophageal reconstruction in children using colon. Arch Dis Child.

[2] Longino LA, Woolley MM, Gross RE (1959). Esophageal replacement in infants and children with use of a segment of colon. J Am Med Assoc.

[3] Hopkins WA, Zwiren GT (1963). Colon Replacement of the esophagus in children. J Thorac Cardiovasc Surg.

[4] Spitz L (1984). Gastric transposition via the mediastinal route for infants with long gap esophageal atresia; J Pediatr. Surg.

[5] Hirschl RB, Yardeni D, Oldham K (2002). Gastric Transposition for Esophageal Replacement in Children. Ann Surg.

[6] Reinberg O (2016). Esophageal replacements in children. Ann NY Acad Sci.

[7] Angotti R, Molinaro F, Noviello C (2017). Gastric transposition as a valid surgical option for esophageal replacement in pediatric patients experience from three Italian medical centers. Gatroenterol Rep.

[8] Kunisaki SM, Coran AG (2017). Esophageal replacement. Sem Ped Surg.

[9] Sharma S, Gupta DK (2017). Surgical techniques for esophageal replacement in children. Pediatr Surg Int.

[10] AbouZeid AA, Zaki AM, Radwan AB (2020). Colonic replacement of the esophagus towards standardization of the technique. J Pediatr Surg.

[11] Saleem M, Iqbal A, Ather U (2020). 14 Years' experience of esophageal replacement surgeries. Ped Surg International.

[12] Tannuri ACA, Angelo SS, Takyi P, Silva AR, Tannuri U (2021). Esophageal substitution or esophageal elongation procedures in patients with complicated esophageal atresia Results of a comparative study. J Pediatr Surg.

[13] Estevão-Costa J, Fragoso AC, Campos M (2011). Transhiatal esophagectomy with gastric transposition for esophageal replacement in post-corrosive stricture in children. Acta Med Port.

[14] Awad K, Jaffray B (2017). Oesophageal replacement with stomach A personal series and review of published experience. J Paediatr Child Health.

[15] Spitz L (2009). Gastric transposition in children. Sem Ped Surg.

[16] Tannuri U, Maksoud-Filho JG, Tannuri ACA, Andrade W, Maksoud JG (2007). Which is better for esophageal substitution in children, esophagocoloplasty or gastric transposition A 27-year experience of a single center. J Pediatr Surg.

[17] Burgos L, Barrena S, Andres AM (2009). Colonic interposition for esophageal replacement in children remains a good choice 33-year median follow-up of 65 patients. J Pediatr Surg.

[18] Bludevich BM, Kauffman JD, Smithers CJ (2020). 30-Day Outcomes Following Esophageal Replacement in Children A National Surgical Quality Improvement Project Pediatric Analysis. J Surg Research.

[19] Bradshaw CJ, Sloan K, Morandi A (2018). Outcomes of esophageal replacement Gastric pull-up and colonic interposition procedures. Eur J Pediatr Surg.

[20] Spitz L, Kiely E, Pierro A (2004). Gastric Transposition in Children - A 21-Year Experience. J Pediatr Surg.

[21] Tannuri U, Tannuri ACA, Goncalves MEP, Cardoso SR (2008). Total gastric transposition is better than partial gastric tube esophagoplasty for esophageal replacement in children. Dis Esophag.

[22] Chávez-Aguilar AH, Silva-Baez H, Sanchez-Rodriguez YB (2015). Early complications with colon esophageal substitution for children via retrosternal. Gac Med Mex.

[23] Tannuri U, Tannuri ACA (2011). Should patients with esophageal atresia be submitted to esophageal substitution before they start walking. Dis Esophag.

[24] Lima M, Destro F, Cantoni M (2015). Long-term follow-up after esophageal replacement in children 45-Year single-center experience. J Pediatr Surg.

[25] Foster JD, Hall NJ, Keys SC, Burge DM (2018). Esophageal replacement by gastric transposition A single surgeon's experience from a tertiary pediatric surgical center. J Pediatr Surg.

